# Quality improvements in diabetes care, how holistic have they been? A case-study from the United Kingdom

**DOI:** 10.1186/1475-9276-13-29

**Published:** 2014-04-15

**Authors:** Emma Wilkinson, Gurch Randhawa, Maninder Singh

**Affiliations:** 1 Institute for Health Research, University of Bedfordshire, Hitchin Road, Luton LU2 8LE, UK

**Keywords:** Type 2 diabetes mellitus, South asian, Patient experience, Diagnosis, Quality, Access, Care pathway

## Abstract

**Aims:**

As quality in diabetes care includes patient centred support for self-management, investigating patients’ experiences upon diagnosis can help improve access to this element of care among diverse populations. This research explored this care in the context of recent national quality improvement initiatives which support self-management.

**Methods:**

South Asian and White European patients over 16 years with a recent (< 1 year) diagnosis of diabetes were recruited from 18 General Practitioner (GP) practices in three UK locations - Luton, West London and Leicester. A semi-structured qualitative interview was conducted with 47 patients.

**Results:**

Twenty one out of 47 (45%) reported unmet support and information needs at diagnosis. Although there was a small proportion of participants (8 out of 47, 17% of all respondents) who felt they did not require any help or support with managing their diabetes because their GP had provided comprehensive and efficient care, there was an equal number who voiced a negative view of the care they had received to date. This concerned information giving, support and communication, suggesting that recently implemented national quality improvement interventions may not have been successful in improving all aspects of diabetes care, particularly those encouraging self-management. The emerging analysis led to consideration of concordance as an important concept through which to understand inequalities and improve access to quality diabetes care. In order to encourage self-management from the start, care providers need to be cognisant that patients are not homogeneous and be responsive to their different information needs and emotional responses to diagnosis.

**Conclusions:**

In order to support self-management and deliver patient centred care in diverse populations, care providers will need to be adaptable to individual needs around diagnosis.

## Introduction

The rising prevalence of diabetes in the UK [1] is well documented. We know that the South Asian population have a higher relative risk of diabetes and kidney complications [2,3] because of genetic, lifestyle or access factors [4,5]. Research to stratify and understand populations based on ethnicity can help us understand better the full extent of the challenge of Type 2 Diabetes Mellitus for those who commission or provide diabetes care [6,7], particularly as ethnic minorities are predicted to make up approximately 43% of the UK population by 2056 [8]. Previous research has also shown that there can be barriers to healthcare access that relate to ethnicity or culture, which culturally competent healthcare providers seek to mitigate [9,10] in order to provide equitable access and quality in diabetes care [11].

Quality care in diabetes includes support for people in managing their diabetes and was part of Standard 3 of the UK National Service Framework: ‘empowering people with diabetes’ through engagement and partnership in decision making [12]. In exploring access to quality diabetes care, this aspect needs to be considered along with access to other supporting interventions such as patient education^a^ and against the policy backdrop to encourage patient centred care and joint care planning [13]. The combination of quantitative and qualitative data which capture the processes of care and the experience of people who live with this complex chronic disease have the potential to help all stakeholders understand how to maximise access to effective diabetes prevention and management.

The fundamental role that people with diabetes play in their own personal management continues to be emphasised through current policy guidance [14,15]. This is not purely in terms of increasing patient empowerment and improving experience, but also because of the potential cost savings [16] in the face of increasing diabetes prevalence rates and costs in the UK [17] of managing diabetes [18] and complications, particularly in ethnic minorities, who have a higher risk of diabetes and end stage renal failure compared to White Europeans [19].

Earlier studies have shown that by the time they are diagnosed with diabetes, half the people already have signs of complications and the onset of diabetes may occur 5–6 years and 10 years prior to diagnosis respectively [20,21]. These highlight the importance of early diagnosis and care for on-going self-management which quality initiatives through the first decade of the new millennium sought to implement [12,13,22].

The Diabetes Care Pathway Study compared access and experience of diabetes care in White European and South Asian groups at three locations in the UK: Leicester, Luton and West London. By using mixed methods and applying a care pathway design to the study we were able to explore and make some evaluation of the impact of the quality initiatives introduced, as well as making suggestions for whom and how diabetes care could be further improved.

This paper describes patient experience early on in the pathway around the period of diagnosis, which is an important time for people to receive information about the condition in an appropriate and individual centred way [12,13,23]. Current policy drivers for patient experience work [24] support the use of patient experience data to improve quality of services in the evolving NHS through attention to the key elements of quality, access, cultural sensitivity, shared decision making, information and communication amongst other patient centred objectives. Patient experiences of diabetes diagnosis and care in 2007, following the introduction of quality improvement initiatives include: national service frameworks for diabetes and kidney disease and the Quality Outcomes Framework (QOF). These are presented and discussed in relation to improving access to quality diabetes care. Broad recommendations are made for NHS diabetes care.

## Methods

The Diabetes Care Pathway Study was implemented at 3 sites: Leicester, Luton and West London (Ealing) through 2006 to 2008. The inclusion of study sites was based on the socio-demographics of the local population to enable the inclusion of patients and providers to patients from the predominant South Asian population groups in the UK i.e.: Indian Gujarati; Indian Punjabi; Pakistani and Bangladeshi.

The overarching study combined audits at two stages in the diabetic renal disease care pathway: at diabetes diagnosis and at referral to specialist renal services in 2004 and 2007, with qualitative interviews from patients and care providers in 2007. This paper is concerned with the interviews of patients who made up the audit sample of people diagnosed with type 2 diabetes in 2007. The audit element has been previously reported [25,26].

### Sample selection and recruitment

The patients for interview were recruited by participating general practitioners who themselves had been recruited to provide a representative sample in terms of practice population demographics, size, type of practice and QOF achievement scores. The sample selection of 18 GP practices was purposive and pragmatic, and the analysis and interpretation of results takes into account study limitations and external validity in relation to sample selection.

It was estimated that up to 20 patients (10 White and 10 South Asian) would be recruited at each site (up to 60 in total), and that this would collectively provide: a representative sample of newly diagnosed patients across the practices, enable at least one patient to be recruited from each participating practice who would be considered an adequate sample for the proposed analysis [27,28] and fit within the resource limitations of the study.

Primary care practice staff conducted a search of their practice database in 2008 to identify patients who fulfilled the inclusion criteria (< 16 years, of White European or South Asian ethnicity and diagnosed with T2DM between 1^st^ January – 31^st^ December 2007). All patients who fulfilled the criteria were sent a letter with a response slip, patient information sheet and stamped addressed envelope for response. Those who returned the response slip indicating that they were willing to take part in an interview received a telephone call from one of the research team (including bilingual researchers as required) to answer any questions the potential participant might have and - if the individual still wished to take part - to arrange the interview.

### Interview format

A semi-structured questionnaire schedule was developed specifically for the purpose of this study. This was devised by collaborating researchers (social scientists and clinicians) and comprised a series of questions with prompts covering the following broad areas: diagnosis of diabetes; symptoms; access to and experience of diabetes services; current health; self-management and support; access to information and communication. Both the patient information sheet and the preamble to the interview asked patients to recount, in their own words, their experience and the interview schedule was intended to be used as a guide and to ensure the main areas were covered during the course of the dialogue. One to one interviews were conducted by researchers in the patient’s preferred venue, invariably the patient’s home, and in the patient’s language of choice, employing bilingual researchers where required. Interviews lasted between 40 minutes to one hour and were tape recorded. The resulting recordings were transcribed verbatim into Microsoft Word documents.

### Analysis

Interview transcripts were repeatedly read through, an initial framework of key themes (initial thematic categories) was formulated and interviews were analysed using these themes as well as others as they emerged. Although broad interview areas had been determined a priori, these themes were identified retrospectively through the analysis process. Analysis of data from these themes forms the basis of the following results and discussion.

**Figure 1:**
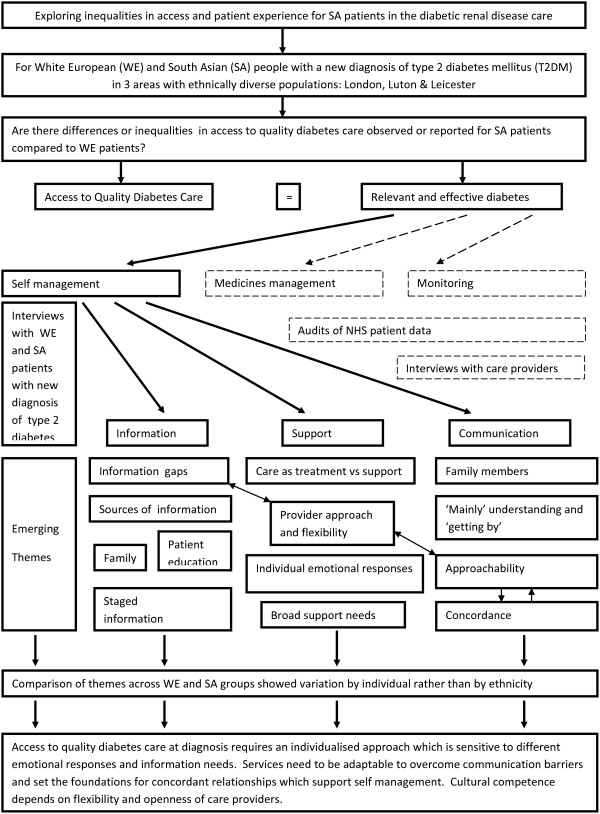
Schematic framework for the research, areas explored and emerging themes in the Diabetic Care Pathway Study.

Figure 1 provides a schematic framework for the research, areas explored and emerging themes in this element of the Diabetes Care Pathway Study. The bold boxes and arrows concern the research question, themes and emerging subthemes discussed in this paper whereas the dashed boxes and arrows relate to other elements of the Diabetes Care Pathway Study which are described elsewhere [9,25,26].

Thematic analysis is a widely used process in the analysis of qualitative data [29,30] and was used in this research to identify a framework of themes and sub themes which relate to the access and quality of healthcare. Nvivo 7, a computer software package for qualitative research, was used to facilitate data coding and retrieval. The lead researcher (EW) conducted the coding and data analysis in collaboration with bilingual research interviewers who provided feedback on individual interviews and the chief investigator (GR), who had oversight of the process. The researchers who carried out the analysis have a background in public health research and in qualitative methods. They were part of a larger interdisciplinary team made up of General Practitioners, Nephrologists and Epidemiologists who assisted in interpreting findings. The interview quotes have been reproduced verbatim and have been made anonymous and attributed to the different groups of participants that were interviewed – SAF = South Asian Female; SAM = South Asian Male; WEF-White European Female; WEM = White European Male.

## Results

Forty-seven people (28 South Asian, 19 White European) with a recent (< 1 yr) diagnosis of T2DM were recruited and interviewed. South Asian participants were 12 years younger at diagnosis (mean age 50.6 years, range 34–77 years) compared to White European participants (means age 63.3 years, range 43–85 years).

The interview sample included slightly more men (4.7%) than women, relative to the proportion within the original audit sample. Recruitment rates were 17.4% and 12.7% respectively and the proportion of White European to South Asian was similar: 59.6% v 54.1% South Asian and 40.2% v 45.9% White European respectively to the original audit sample.

### Information

Twenty-one (44.7%) of people reported having information needs in various ways – diet detail; risk and complications explained; what monitoring to expect:

#### Information gaps

*‘Well, I have never been told anything about the things that I have said; like to see a dietician or eye checks, to be given an idea of what sort of symptoms I can expect over the years.’***WEF1**

*‘They don’t really tell you what would happen if it carried on…and I think that’s important to know. But the only way we know is because my brother-in-law is a doctor and he told us…otherwise we wouldn’t have known; so I think it’s important to let people know.’***SAF1**

#### Sources of information

For many of the respondents, family members were a good source of information. In regards to awareness about diabetes, only a very small number (n = 1 out of 28) had received structured patient education at two of the three sites. At the third, nearly half of the participants had either attended the DESMOND^b^ programme or a shorter locally developed alternative for non-English speakers. Those who had attended the DESMOND sessions suggested that the amount and timing of information was problematic for people recently diagnosed with diabetes, and that in retrospect they might have found it more useful for education to be delivered in a more staggered way:

#### Staged information

*‘It was an awful lot of information to take in. I mean, they did say how the pancreas doesn’t work and it affects the liver and - but there’s a lot there I’m sure they didn’t take in, because the older you get the less you retain anyway. Well, I do. I find I have to do things in the mornings.’***WEF1**

*‘I think when you’re first diagnosed it does come as a shock and you think, oh, you know, then you sort of - oh it can’t be me - and then you’ve got all this information in one day and you just don’t take it all in, you know. I think maybe it’s better 6 months down the line when you’ve sort of complied, you know, sort of, you’ve got it and then go. I mean, I could have done with this sort of say 6 months ago when I’d got it into my head that, you know, I need to sort of look after myself a bit better, you know.’***WEF2**

### Support

Reflection on their experiences during the first year of diagnosis also prompted participants to comment on the care provided by their GP in terms of support. A small number reported their satisfaction with the quality of the care they had received to date, whilst a similar number described their dissatisfaction. The following extracts illustrate these contrasting experiences and opinions of diabetes care:

### Care as treatment versus support

*‘So as far as I am concerned about diabetes it was found out rather quickly, it was treated rather quickly and professionally.*’ **WEM1**

*‘At present my Doctor is excellent, I am able to talk to him and ask questions whereas the previous one I couldn’t. Initially, I did not ask for any help as my GP had a bad, unapproachable attitude.’***SAM1**

#### Provider approach and flexibility

*‘The nurse is very good and the GP was great around diagnosis but of late has not been so supportive. I want to work with a non-medical route more but my GP wants me to take medication so it is difficult. I wish I could have a discussion or partnership but it is hard to speak to him…I understand the long term consequences of not taking medication, I am painfully aware of the implications but I would like to give it a chance to work…use different ways of dealing with it, perhaps not such a standardised route. Doctors just see you as one of many, do not cater for individuals.’***WEM2**

*‘I don’t feel they support you as such. I think they just follow protocol. It’s like a standard procedure they go through. They give you leaflets and stuff but it is up to you to read, they are not concerned if you do or not. As far as they are concerned they have done what they have to and then it is up to you; which I kind of understand but I don’t find it very helpful…The GP just checks me out and does the routine stuff but does not give me any encouragement nor does he understand what I am going through. I suppose the GP and nurse are just doing their job, I don’t think they are bothered.’***SAM2**

#### Comparison by ethnicity

A comparison of responses across the White European and South Asian groups of respondents found that the patient experiences varied by individual rather than being common experiences for either ethnic group. This is illustrated by the following extracts from interviews with three people who share the same broad SA ethnicity grouping but had quite different emotional responses to diabetes diagnosis:

#### Individual emotional response

*‘And I was so upset; and she said: “he mentioned you will be on insulin”. That was the first time I went when I was doing my blood test. And I complained, I said, “I don’t want it”…I had to make an appointment, because I was so scared thinking: “oh my God, what am I supposed to eat now?” I was confused. So one of the nurses said: “oh just eat normally except no sugar and less butter and…”, because any fat, because it’s (inaudible) if you’re diabetic that can give, what do you call it, harden arteries and give you a heart attack.’***SAF4**

*‘No, no, I don’t put in the mind to the diabetes, because if you put in your mind you get more worse. So some people have cancer, like, so every day they put in the mind “oh I have cancer” and second, third day…so what’s wrong with that, one day is going to crash. If you’re cured, that’s a cure; it’s good; it’s in the mind. ‘***SAM3**

*‘So yeah, you’ve got to keep it in perspective, you know, information is a good thing but sometimes it can be a bad thing in the sense it causes more anxiety. So as long as, you know, you keep it in a balanced sort of way, keep it in perspective, then at least you’ll understand it; that’s the main thing. Because it’s no use ignoring it, you know, being like an ostrich, digging your head in the sand thinking “oh, none of these things will happen”, you have to, you know, accept it, once you’ve got diabetes there are certain things that will sort of happen but those things can be prevented as far as possible by you carrying out these measures.’***SAM4**

This analysis also identified some of the broad support needs reported by individuals and related to access and patient centred care. For older participants, there were other pressing concerns at diabetes diagnosis such as issues of accommodation and mobility:

#### Broad support needs

*‘These are the two things I am most concerned about it, because in my situation…I want to have good accommodation, you know, that I am suggesting really, backing it to the city council that I needed a bungalow where I can have it a little garden and I can do little bit gardening…You know, being independent I like it, that sort of thing, but that is a suggestion, not for diabetics but for me this is, I just wanted my own - but thing is…they won’t do it. They can do it for people who are getting worse, for them they can put it very quickly for accommodation, but I am getting, you know, controlling myself better and I am out of their list.’***SAF5**

*‘Yeah, I’d like help yeah, because I can’t walk, I told them because I can’t walk I need something supporting me and he said, “you not need, instead of holding something to walk ….’***SAM3**

### Communication

Although a comparison of responses across the two ethnic groups found that differences were individual rather than related to ethnicity, when it came to communication more than a third of the SA participants (9 out of 25, 19% of all respondents) reported having to rely on family members to translate information during clinical encounters:

#### Family members

*‘As I can’t speak the language myself, I always take my brother with me. But I do ask questions through him, like what to do and when.’***SAF2**

#### ‘Mainly’ understanding and ‘getting by’

*‘Even though I don’t speak English fluently I understand and manage to get by. Also, my GP is very good and explains well to me.’***SAF3**

This may have affected their access to aspects of care which concern communication between patients and care providers as these respondents reported that they ‘mainly’ understood what was said during clinical encounters, no-one reported interpreters assisting with appointments and only a small number of the SA respondents said the GP spoke their language.

## Discussion

The findings of this study are of benefit to health care commissioners and providers around the world who look after multi-ethnic populations: 44% of participants in this study had information (support) needs which had not been met at diagnosis despite other improvements in quality of care prior to 2007, such as improved monitoring [25,26]. This represents a missing link in providing patient centred care within a chronic disease model for diabetes in primary care [31] and missed opportunities for information exchange.

For patients to have access to quality care for improved health outcomes, that care must be relevant and effective [11]. The role of healthcare providers in facilitating access to diabetes care includes the provision of meaningful information to support patients to make decisions about their own care [13], amongst other activities such as the prescription of medication and monitoring of the condition (Figure 1).

Engagement with their GP and practice nurse is the means by which most of diabetes care is accessed by patients thus building of a concordant relationship from diagnosis onwards is vital in supporting this. In addition, the time around diagnosis is an important period for engaging with patients in a positive way to minimise stress/distress [32] and to provide information, answer questions and voice concerns [23,33].

This study interviewed patients within a year of diabetes diagnosis but were at different points within that first year. Therefore, the data collection was unlikely to have captured all patients’ experiences as some patients were yet to receive additional care. The information needs may have therefore have reduced by the end of the year. However, it is equally possible that different information and support needs may have been identified and other barriers to access may have become apparent by the time one year had passed.

At two of the sites only a very small number of respondents had received any structured diabetes education at the time they were interviewed, despite guidance for providers which promoted this as a means of meeting the diabetes quality standards [34]. Diabetes education is a cornerstone of an empowerment for effective diabetes self-management and our data seems to suggest that in our sample in 2007 there was variable access to this element of care in the period following diagnosis. As general practitioners act as gatekeepers to diabetes education through referral of patients they play an important role in ensuring patient access to that aspect of diabetes care.

Whilst it has been questioned whether an empowerment approach is in itself appropriate for all cultures [35] it is universally agreed that for self-management to be supported there needs to be collaboration and communication between patient and healthcare provider from the outset and there on afterwards [13,36]. Furthermore, the concept of patient centred care is based on premise that services are aware of and able to respond to the specific needs of patients in relation to their condition, and for this to occur there must be effective communication between care provider and patient.

The novel approach of the Diabetes Care Pathway Study was that it explored patient experience and quality of diabetes from a realist and holistic perspective following implementation of national policy and quality initiatives. The novel findings from the qualitative element reported here are that they suggest - within the context of quality improvements in detection, management and monitoring - the fundamental process of concordance to facilitate and encourage self-management is a matter of meaningful interactions between individuals - that is, patients and their care providers.

The comparisons by broad ethnic categories emphasised this by showing individual differences rather than generic cultural ones in response to diabetes diagnosis and care. This is not to say that culture is not important in understanding both the delivery of diabetes care and patient attitudes to it, but that our results suggest that quality improvements should improve access at the level of person to person interaction and cultural competency through provider communication, openness and approachability.

Patients who were critical of the care they had received identified that recognition of individual needs and open communication to discuss these by GP’s were lacking. Unless there is two-way communication in diabetes care, it is unlikely that the care provider is able to gauge individual response to diagnosis and therefore support self-management early on in a meaningful and timely way. Others have found that there is a lack of content and effective communication within interactions between GPs and non-fluent South Asian patients, even though consultations tend to be longer [37]. This may be indicative of less informative as well as less meaningful and therefore less concordant interactions in consultations when language is a barrier.

More than a third of the South Asian participants (9 out of 25, 19%) reported having to rely on family members to translate information. If this is the case in our sample of willing respondents, we can only surmise that for others who may be less health literate or not have access to bilingual communication about their health, lack of information and support at diagnosis, this may be an even bigger issue. Whether ‘mainly’ understanding what care providers are saying is satisfactory in the context of supporting self-management of T2DM is a question these results raise. Further work is required to find effective communication methods for supporting South Asian patients who are not fluent in English with this aspect of diabetes care.

Future demographic shifts towards greater proportions of older people [38] and ethnic minorities in the UK population (8), together with predicted rises in obesity and related chronic diseases, make support for effective self-management of T2DM all the more pressing. This qualitative exploration of the experiences of White European and South Asian patients recently diagnosed with diabetes found few issues which related directly to respondents’ ethnicity, but highlighted some of the universal challenges for care providers in ensuring access to quality diabetes care for all patients.

The range of experience expressed by this sample of people with a new diagnosis of diabetes indicates that a one size is not likely to fit all when it comes to supporting patients in self-management early on in the care pathway. Patients who talked about poor communication with their GP identified that it varied with the individual clinician, but a standardised approach which did not extend to good patient-practitioner communication lacked individual support for diabetes self-management. As quality improvement guidance focussed on patient empowerment as a main objective and patient centred care has been an overarching theme in NHS policy for some time, it seems that this element of diabetes care has been more difficult for primary care to implement compared to others such as monitoring [25,26].

This qualitative study seeks to understand some of the processes of quality diabetes care from the patient’s perspective in a sample of people recently diagnosed with diabetes. Non-responders were not followed up and it is possible that the people interviewed had better access in the first place, particularly with respect to the South Asian population for whom there can be additional barriers to taking part in research [39]. The observations of the range of individual experience in diabetes diagnosis and care - as well as the unmet information and support needs during the first year - may therefore be greater in the wider population. A primary care workforce that can be adaptable to communication and work with a range of reactions, knowledge and attitudes towards diabetes diagnosis has the potential to encourage effective diabetes self-management in the future.

Our analysis seems to show that, although many people were satisfied with the diabetes care they had been receiving, their needs with respect to information and support had not yet been met and that communication with providers plays an important role. The facilitation of self-care by the primary care team appears to be the more difficult element of diabetes care to implement as it is necessarily individualised and requires the resources of time and communication to build a concordant relationship.

Finally, learning from accounts of individual people’s concerns and priorities regarding their diabetes care, a more holistic social model of patient centred care and access is required to be adopted in primary care. This should encompass wider issues such as mobility and environments, and respond to the changing needs and priorities of people with diabetes as they grow older.

### Limitations

The patient sample came from an audit of all patients diagnosed with T2DM during 2007 at 18 GP practices in Leicester, Luton and West London that had been purposively recruited to include the variety of GP practices at those locations.

Potential interviewees were approached by letter and non-responders were not followed up, so it is possible that the interview sample achieved was not representative of the audit population. Ethnic minority groups tend to be under represented in clinical health research and it is possible that we did not recruit patients who are less engaged with the health system and who experience the most barriers to access. This could be the case for both the groups in this research and, as written recruitment literature was used in the first instance and was not translated from English, there was a potential barrier for people unable to read English.

The study was exploratory in that it considered ethnicity, which in itself is a complex concept. It was not possible, within the constraints of size and resources together with incomplete and inaccurate recording of ethnicity in primary care, to achieve a sample which was able to fully explore comparisons based on ethnicity over and above the broad South Asian and White European categories used in this study.

Although interviews were conducted in the participant’s preferred language, by bilingual interviewers where necessary in order to obtain quality data, it is possible that there was some bias through translation. Many of the interviews and all the thematic analysis were conducted by an experienced researcher but bias could also have occurred through misinterpretation.

Low participation rates therefore may have been due to research and context specific factors such as the initial recruitment method (postal rather than face to face or by telephone) and the fact that potential participants who had recently been diagnosed with diabetes may have felt they had limited experience of diabetes care to report. However, the relative proportion of South Asian and White European respondents was similar to the audit sample 58% vs 59.6% South Asian and 42% vs 40.2% White European so there appeared to be no particular bias in participation by either group notwithstanding other barriers to participation [40].

## Conclusions

Our study shows that care varied for people with recent diabetes diagnosis at the three study sites, as not all recently diagnosed patients had access to all aspects of quality diabetes care and therefore were met with a lack of support and information. There was evidence that deficits in care at diabetes diagnosis related to GP communication, information giving and support. The findings highlight that access to care for South Asian patients was affected if they were not fluent in English and had to rely on family members to translate. The quality improvement initiatives had not achieved access to quality diabetes care for all respondents. In order to support self-management and deliver patient centred care in diverse populations, health care commissioners and care providers will need to be adaptable to individual needs around diagnosis.

## Endnotes

^a^Patient education was defined by the UK National Institute for Clinical Excellence in 2005 as: ‘a planned and graded programme that is comprehensive in scope, flexible in content, responsive to an individual’s clinical and psychological needs, and is adaptable to his or her educational and cultural background.’ [41].

^b^DESMOND - Diabetes Education and Self-Management for Ongoing and Newly Diagnosed [42].

### Ethical approval

This study was given ethical approval by Bedfordshire NHS Research Ethics Committee in June 2005 - REC reference number: 05/Q0202/24.

## Competing interests

The authors declare that they have no competing interests.

## Authors’ contributions

EW carried out the fieldwork, undertook analysis and drafted paper; GR secured funding for the study, designed the study and assisted with drafting paper; MS assisted with drafting paper. All authors read and approve the final manuscript.

## References

[B1] Diabetes UK Diabetes in the UK 2011/2012 Key statistics on diabetes 2011 [http://www.diabetes.org.uk/Documents/Reports/Diabetes-in-the-UK-2011-12.pdf]

[B2] Roderick P Raleigh V Hallam L Mallick N The need and demand for renal replacement therapy in ethnic minorities in England J Epidemiol Community Health 1996 50 334 339 10.1136/jech.50.3.334 8935467 PMC1060292

[B3] Burden A McNally P Feehally J Increased incidence of end stage renal failure secondary to diabetes mellitus in Asian ethnic groups in the United Kingdom Diabet Med 1992 9 641 645 10.1111/j.1464-5491.1992.tb01860.x 1511571

[B4] Raleigh V Diabetes and hypertension in Britain’s ethnic minority communities: implications for the future of renal services Br Med J 1997 314 209 212 10.1136/bmj.314.7075.209 9022442 PMC2125665

[B5] Johnson M Owen D Blackburn C Black and minority ethnic groups in England: The second health and lifestyles survey 2000 London: Health Education Authority

[B6] Riste L Khan F Cruickshank K High prevalence of type 2 diabetes in All ethnic groups, including Europeans, in a British inner city Diabetes Care 2001 24 8 1377 1383 10.2337/diacare.24.8.1377 11473073

[B7] Verma A Birger R Bhatt H Murray J Millett C Saxena S Banarsee R Gnani S Majeed A Ethnic disparities in diabetes management: a 10-year population-based repeated cross-sectional study in UK primary care J Public Health 2010 32 2 250 258 10.1093/pubmed/fdp114 20064875

[B8] Coleman D Projections of the ethnic minority populations of the united kingdom 2006 2056 [https://www.spi.ox.ac.uk/fileadmin/documents/PDF/WP55_Projections_of_the_Ethnic_Minority_populations_of_the_United_Kingdom.pdf] 10.1111/j.1728-4457.2010.00342.x 20882702

[B9] Wilkinson E Randhawa G Concordance facilitates access in diabetes care – service provider perspectives of service improvement and cultural competency Diabet Med 2012 29 1440 1446 10.1111/j.1464-5491.2012.03674.x 22486243

[B10] Betancourt J Green A Carrillo J Ananeh-Firempong O Defining cultural competence: a practical framework for addressing racial/ethnic disparities in health and healthcare Public Health Rep 2003 118 293 302 10.1016/S0033-3549(04)50253-4 12815076 PMC1497553

[B11] Gulliford M Figueroa-Munoz J Morgan M Hughes D Gibson B Beech R Hudson M What does ‘access to health care’ mean? J Health Serv Res Po 2002 7 186 188 10.1258/135581902760082517 12171751

[B12] Department of Health National service framework for diabetes: standards 2001 London: HMSO [http://webarchive.nationalarchives.gov.uk/20130107105354/http://www.dh.gov.uk/en/Publicationsandstatistics/Publications/PublicationsPolicyAndGuidance/DH_4002951]

[B13] Department of Health Care planning in diabetes 2006 London: HMSO

[B14] National Institute for Health and Clinical Excellence Patient experience in adult NHS services: improving the experience of care for people using adult NHS services Clinical Guideline 2012 138 [http://www.nice.org.uk/nicemedia/live/13668/58284/58284.pdf] 34260162

[B15] National Institute for Health and Clinical Excellence Type 2 diabetes: the management of type 2 diabetes Clinial Guideline 2008 66 [http://www.nice.org.uk/nicemedia/pdf/CG66NICEGuideline.pdf]

[B16] Loveman E Cave C Green C Royle P Dunn N Waugh N The clinical and cost effectiveness of patient education models for diabetes: a systematic review and economic evaluation Health Technol Assess 2003 7 22 1 190 [http://www.ncbi.nlm.nih.gov/pubmedhealth/PMH0015175/pdf/summ722.pdf] 10.3310/hta7220 13678547

[B17] Diabetes UK The state of the nation 2012 [https://www.diabetes.org.uk/Documents/Reports/State-of-the-Nation-2012.pdf]

[B18] Currie C Gale A Poole C Estimation of primary care treatment costs and treatment efficacy for people with type 1 and type 2 diabetes in united kingdom from 1997–2007 Diabet Med 2010 27 938 948 10.1111/j.1464-5491.2010.03040.x 20653753

[B19] Randhawa G Renal health and transplantation – a focus on ethnicity J Ren Care 2012 38 109 114 22348370 10.1111/j.1755-6686.2012.00277.x

[B20] Group UKPDS UK prospective diabetes study VIII: study design, progress and performance Diabetologia 1991 34 12 877 90 1778353

[B21] Harris M Klein R Welborn T Knuiman M Onset of NIDDM occurs at least 4–7 years before clinical diagnosis Diabetes Care 1992 15 7 815 819 10.2337/diacare.15.7.815 1516497

[B22] Department of Health National service framework for diabetes delivery strategy 2002 [http://webarchive.nationalarchives.gov.uk/20130107105354/http://www.dh.gov.uk/en/publicationsandstatistics/publications/publicationspolicyandguidance/browsable/DH_4899696]

[B23] Peel E Parry O Douglas M Lawton J Diagnosis of type 2 diabetes: a qualitative analysis of patients’ emotional reactions and views about information provision Patient Educ Couns 2004 53 269 275 10.1016/j.pec.2003.07.010 15186863

[B24] NHS Institute for Innovation and Improvement Transforming patient experience: the essential guide – the policy frameworhttp://www.institute.nhs.uk/patient_experience/guide/the_policy_framework.html

[B25] Wilkinson E Randhawa G Rehman T Abubacker T The impact of quality improvement initiatives on diabetes care among South Asian people Diabetes and Primary Care 2011 13 2 90 98

[B26] Wilkinson E Randhawa G Roderick P Rehman T Abubacker T National primary care guidelines for England: impact on chronic kidney disease prevention in South Asian populations J Nephrol 2012 25 5 661 71 10.5301/jn.5000037 21983987

[B27] Guest G Bunce A Johnson L How many interviews are enough? An experiment with data saturation and variability Field Method 2006 18 1 59 82 10.1177/1525822X05279903

[B28] Pope C Ziebland S Mays N Analysing qualitative data Br Med J 2000 320 114 116 10.1136/bmj.320.7227.114 10625273 PMC1117368

[B29] Braun V Clarke V Using thematic analysis in psychology Qualitative Research in Psychology 2006 3 77 101 10.1191/1478088706qp063oa

[B30] Ritchie J Lewis J Qualitative research practice. a Guide for social science students and researchers 2008 London: SAGE

[B31] Bodenheimer T Wagner E Grumbauch K Improving primary care for patients with chronic illness. The chronic care model, part 2 JAMA 2002 288 15 1909 1914 10.1001/jama.288.15.1909 12377092

[B32] Wilkinson E Randhawa G Singh M What’s the worry with diabetes? learning from the experiences of white European and south Asian people with a new diagnosis of diabetes Prim Care Diabetes 2013 [http://dx.doi.org/10.1016/j.pcd.2013.11.006] 10.1016/j.pcd.2013.11.006 24361373

[B33] Lawton J Parry O Peel E Douglas M Diabetes service provision: a qualitative study of newly diagnosed type 2 diabetes patints’ experiences and views Diabet Med 2005 22 1246 1251 10.1111/j.1464-5491.2005.01619.x 16108856

[B34] Diabetes UK, Department of Health Structured patient education in diabetes: report from the working group 2005 http://www.diabetes.org.uk/Documents/Reports/StructuredPatientEd.pdf

[B35] Asimakopoulou K Scramber S Newton P First do no harm’ the pitfalls and stumbling blocks of empowermen European Diabetes Nursing 2010 7 2 79 81 10.1002/edn.162

[B36] Funnell M Anderson R Empowerment and self-management of diabetes Clinical Diabetes 2004 22 3 123 127 10.2337/diaclin.22.3.123

[B37] Neale D Ali N Atkin K Alleger V Ali S Coleman T Communication between south Asian patients and GPs: comparative study using he roter interactional analysis system Br J Gen Pract 2006 56 869 875 17132355 PMC1927096

[B38] UK National Statistics Older people[http://www.statistics.gov.uk/hub/population/ageing/older-people]

[B39] Lloyd C Douglas J, Earle S, Handsley L, Lloyd C, Spurr S Researching the views of diabetes service users from South Asian backgrounds: a reflection on some of the issues A reader in promoting public heatlh – challenge and controversy 2010 2 Sage Publications

[B40] Rooney L Bhopal R Halani L Levy M Partridge M Netuveli G Car J Griffiths C Atkinson J Lindsay G Sheikh A Promoting recruitment of minority ethnic groups into research: qualitative study exploring the views of South Asian people with asthma J Public Health 2011 33 4 604 15 10.1093/pubmed/fdq100 21228023

[B41] National Institute for Clinical Excellence Guidance on the use of patient education models for diabetes: technology appraisal 60 2003 [http://www.nice.org.uk/nicemedia/pdf/60Patienteducationmodelsfullguidance.pdf]

[B42] DESMOND [http://www.desmond-project.org.uk/aboutus-269.html]

